# Efficiency of Constructed Wetland Vegetated with *Cyperus alternifolius* Applied for Municipal Wastewater Treatment

**DOI:** 10.1155/2013/815962

**Published:** 2013-08-20

**Authors:** Asghar Ebrahimi, Ensiyeh Taheri, Mohammad Hassan Ehrampoush, Sara Nasiri, Fatemeh Jalali, Rahele Soltani, Ali Fatehizadeh

**Affiliations:** ^1^Department of Environmental Health, Shahid Saddooghi University of Medical Sciences, Yazd, Iran; ^2^Environment Research Center, Isfahan University of Medical Sciences (IUMS), Isfahan, Iran; ^3^Department of Environmental Health Engineering, School of Health, Student Research Center, IUMS, Isfahan, Iran; ^4^Department of Health Education and Promotion, School of Health, Student Research Center, IUMS, Isfahan, Iran

## Abstract

The treatment of municipal wastewater from Yazd city (center of Iran) by constructed wetland vegetated with *Cyperus alternifolius* was assessed. Two identical wetlands with a total working volume of 60 L and 10 cm sandy layer at the bottom were used. First wetland (W1) was control and had no *Cyperus alternifolius* plant. Second wetland (W2) had 100 *Cyperus alternifolius* shrubs with 40 cm height. Influent wastewater was provided from Yazd's septic tanks effluents and after a 4-day retention time in wetlands, reactors effluent was sampled for parameters analysis. Results show that chemical oxygen demand (COD), NO_3_^−^–N, NH_4_^+^–N, and PO_4_^−3^–P in W1 were reduced to 72%, 88%, 32%, and 0.8%, and in W2, these parameters were removed in values of 83%, 81%, 47%, and 10%, respectively. In both wetlands, the highest and lowest removal efficiencies were related to COD and phosphorus, respectively. Also, the removed phosphorus can be released to stream when the soil saturated or influent phosphorus decreased and when the plant died. After a 4-day-retention time, the W2 wetland showed a statistically significantly lower COD and NH_4_^+^–N in comparison with W2 wetland.

## 1. Introduction 

As the word suggests, a natural wetland is an area of ground that is saturated with water, at least periodically. Plants that grow in wetlands, which are often called wetland plants or saprophyte, have to be capable of adapting to the growth in saturated soil [1]. Wetland plants are an important component of wetlands, and the plants have several roles in relation to the wastewater treatment processes. The ability of wetlands to transform and store organic matter and nutrients has resulted in a widespread use of wetland for wastewater treatment worldwide [2]. Wetlands can be used for primary, secondary, and tertiary treatments of domestic wastewater, storm wastewater, combined sewer overflows (CSF), overland runoff, and industrial wastewater such as landfill leachate and petrochemical industries wastewater [3, 4].

Moreover, the use of constructed wetlands is now recognized as an accepted ecotechnology especially beneficial to small towns or industries that cannot afford expensive conventional treatment systems [5, 6]. The most common systems are designed with horizontal subsurface flow (HF CWs) but vertical flow (VF CWs) systems are getting more popular at present. Constructed wetlands with free water surface (FWS CWs) are not used as much as the HF or VF systems despite being one of the oldest designs in Europe [1, 7]. Constructed wetlands (CWs) for wastewater treatment are potentially a good solution for treating domestic and industrial wastewaters in less-developed countries with warm and tropical climates [8]. The selection of plants is an important matter regarding wetlands. Selected plants must be tolerant to toxicity and the changes in the entering wastewater characters. In Europe, most plants used in wetlands are *Phragmites australis*. However, other species such as *Typha *spp.*, Scirpus *spp., and *Phalaris arundinacea *are also used in wetlands. In Portugal, such species as *P. australis*, *Iris pseudacorus, *and *Cyperus *spp. are used as macrophytes in wetlands [4]. *Umbrella sedge* or *umbrella palm *whose scientific name is *Cyperus alternifolius* is a multi-year-old plant that can be grown in humid soil or in marshy areas. The plant has strong underground root and erect aerial stem which does not have any branches. *Cyperus alternifolius *can be easily multiplied using seeds and pieces of the plant [9]. It has been used in different studies as a wetland plant. *Cyperus alternifolius*' advantage compared with other plants like *Miscanthidium violaceum*is is that it eliminates nutrients of the wastewater [10].

Yazd with the area of 131,551 km² is situated at an oasis where the Dasht-e Kavir desert and the Dasht-e Lut desert meet. The city itself is located at 1203 m. above sea-level, and it covers 16,000 km². The population of the Yazd city was 550,000 at the 2011 census.

Yazd is the driest major city in Iran with an average annual rainfall of only 60 mm, and it is also the hottest north of the persian gulf coast with summer temperatures very frequently above 40°C in blazing sunshine with no humidity.

Given the cost-effectiveness of the wetlands for the purpose of wastewaters treatment and easy planting of *Cyperus alternifolius*, the present research studies this plant as wetland macrophyte under the weather conditions of Yazd, in Iran, for municipal wastewater treatment. In addition, the nutrient removal efficiencies by constructed wetland vegetated with *Cyperus alternifolius* were studied and discussed.

## 2. Materials and Methods

### 2.1. Chemicals and Instrumentation

All the chemicals in this study were of extra pure or analytical grade and prepared from Merck Co. COD, nitrate, ammonia, and phosphorus concentrations in influent and effluent wastewater solutions were measured using a Dr/2000 HACH spectrophotometer (Germany).

### 2.2. Wetland Setup and Tests Procedure

**Figure 1:**
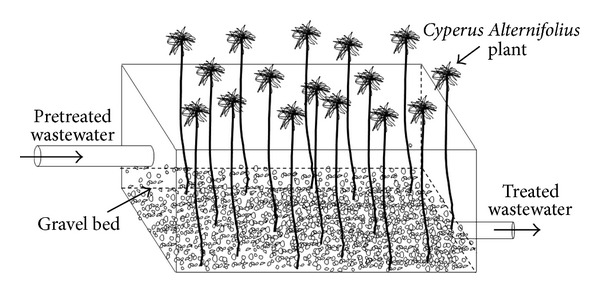
Design of constructed wetland vegetated with *Cyperus alternifolius. *

The present research is an interventional study and surveys the efficiency of the *Cyperus alternifolius *in eliminating the parameters such as COD, nitrate (NO_3_^−^–N), ammonia (NH_4_^+^–N), and phosphorus (PO_4_^−3^–P) in the winter of 2011 in Yazd. Two identical wetlands with a length of 50, a width of 40, and a depth of 30 cm with a total working volume of 60 L were operated in parallel and fed with Yazd municipal wastewater. Sandy layer with a height of 10 cm by various diameters was put at the bottom of the reactor. Larger sands were put near the reactor's tap and the medium and small size sands were put on the middle and the end of the reactor, respectively. Some clay was also added to the reactors. In the main reactor (W2), 100 *Cyperus alternifolius* with an average size of 40 cm were planted.  Figure 1 shows the design and setup of wetland with *Cyperus alternifolius* in a laboratory.

In control wetland (W1), the *Cyperus alternifolius* plants were not cultivated inside W1. The wastewater entering in both reactors was derived from Yazd's septic tank. Average pH and temperature of the incoming wastewater were 8.6 and 16.5°C during the project. At first, 8 L of wastewater was poured to each reactor and the variation of wastewater parameters in outlet after 4 days was examined. The efficiency of *Cyperus alternifolius* in wastewater treatment was studied by comparing the level of parameters in the inlet and outlet of the reactor.

The nominal hydraulic retention time (HRT) is defined as the ratio of the useable wetland water volume to the average flow rate (*Q*_ave_). The theoretical HRT can be calculated as follows:

(1) $$\begin{array}{c} t=\frac{V_{W}\times \epsilon }{Q_{\text{ave}}}, \end{array}$$

where *V*_*W*_ is the volume constructed wetland and *ϵ* is the porosity of a wetland. The wetland porosity values have ranged from 0.65 to 0.75 for fully vegetated wetlands and for dense to less-mature wetlands, respectively [11, 12].

### 2.3. Analysis of Samples

Wastewater samples were analyzed for NO_3_^−^–N, NH_4_^+^–N and PO_4_^−3^–P contents by a spectrophotometer. COD was measured according to the Standard Methods [13].

### 2.4. Statistical Analyses

Tests for significant differences between treatments (various influent parameters and reactors) were determined by a two-sample *t*-test (or Mann-Whitney). Pearson correlation coefficients were used to examine the relationship between the initial concentration of each parameter and related removal efficiency. The results were considered significant when *P* < 0.05. All calculations were performed through the version 18 of SPSS for windows.

## 3. Results

### 3.1. Effect of *Cyperus alternifolius* on COD Removal

**Figure 2:**
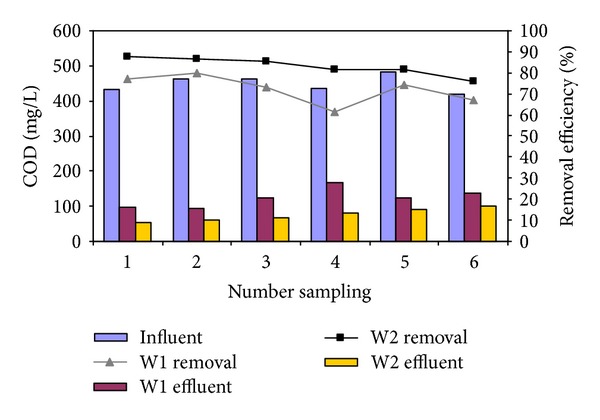
Effect of initial COD concentration on effluent and removal percentage of COD in W1 and W2 (HRT: 42 d and 16.5°C).

The municipal wastewater from Yazd city (Iran) was treated in two identical wetlands as main and control (W2 and W1). The performance of *Cyperus alternifolius* was assessed and analyzed for COD removal in COD concentration of 432, 462, 464, 437, 484, and 420 mg/L corresponding to COD loading of 2.16, 2.31, 2.32, 2.19, 2.42, and 2.1 g/m^2^.d, respectively.  Figure 2 shows the wastewater COD in the different COD concentration and COD loading.

In general, the main reactor exhibited significant (*P* value < 0.05) higher values for the COD removal compared to the control reactor (Table 1). Relatively high correlation was not observed between initial COD concentration and the removal percentage of COD (*r*: 0.28, *P* value > 0.38).

### 3.2. Variation of NO_3_^−^–N Concentration by *Cyperus alternifolius*

To determine the variation of NO_3_^−^–N concentration by *Cyperus alternifolius*, the NO_3_^−^–N removal with initial NO_3_^−^–N concentration of 11, 13.8, 13.9, 14.7, 22.3, and 29.4 mg/L was investigated over NO_3_^−^–N loading of 0.055, 0.069, 0.07, 0.074, 0.112, and 0.147 g/m^2^.d, respectively.

**Figure 3:**
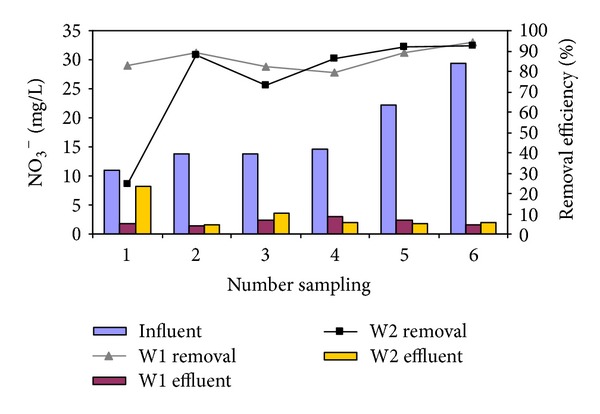
Variation of effluent NO_3_^−^–N concentration in W1 and W2 (HRT: 42 d and 16.5°C).

Figure 3 illustrates the changes of nitrate concentrations by considering influent concentration, loading rate, and reactor type. Over all, Figure 3 shows that the nitrate removal percentage was higher in reactor *by Cyperus alternifolius* (W2) than that without *Cyperus alternifolius* (W1) under the conditions evaluated. The results are depicted in Figure 3, which shows an increase in the nitrate removal efficiency over time with increasing initial nitrate concentration. In W1 and W2, the highest nitrate removal efficiency was obtained at 29.4 mg/L initial nitrate concentration and corresponding to nitrate removal efficiency of 94 and 93%, respectively.

The effluent concentration and removal of NO_3_^−^–N by W1 and W2 were statistically compared using the Mann–Whitney *U* tests (Table 2). The NO_3_^−^–N effluent concentration from *Cyperus alternifolius *wetland (W2) was not significantly higher in comparison with the nitrate effluent concentration from control reactor (*P* value > 0.05). Relatively high correlation was not observed between initial NO_3_^−^–N concentration and NO_3_^−^–N removal (*r*: 0.52, *P* value > 0.08).

### 3.3. Influence of PO_4_^−3^–P Concentration in the Phosphorus Removal

**Figure 4:**
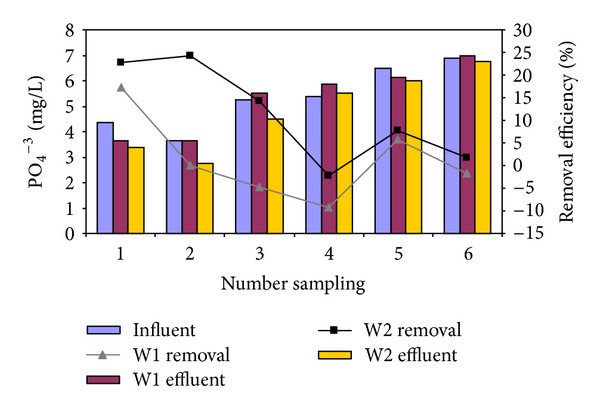
Effluent concentration and removal efficiency of PO_4_^−3^–P as functions of initial concentration (HRT: 42 d and 16.5°C).

The effect of *Cyperus alternifolius* on the phosphorus removal was studied using different initial PO_4_^−3^–P concentrations 4.38, 3.62, 5.25, 5.38, 6.5, and 6.88 mg/L and PO_4_^−3^–P loading of 0.022, 0.018, 0.026, 0.027, 0.033, and 0.034 g/m^2^.d, respectively. The variation of influent and effluent PO_4_^−3^–P concentration and PO_4_^−3^–P removal efficiencies using W1 and W2 are illustrated in Figure 4. As seen in Figure 4, PO_4_^−3^–P removal decreased almost linearly by mounting influent PO_4_^−3^–P concentration. In W1 and W2, as the influent PO_4_^−3^–P concentration increased from 4.4 to 6.8 mg/L, the removal efficiency fluctuated from 17 and 23% to −2 and 2%, respectively.

Table 3 presents the results of average and standard deviation of effluent PO_4_^−3^–P concentration and PO_4_^−3^–P removal efficiency. W1 and W2 were statistically compared using the independent samples *t*-test (Table 3). For each effluent PO_4_^−3^–P concentration and PO_4_^−3^–P removal efficiency, no significant differences between the W1 and W2 were observed (*P* value > 0.05). Relatively high correlation was not observed between initial PO_4_^−3^–P concentration and their removal efficiency (*r*: −0.46, *P* value > 0.13).

### 3.4. NH_4_^+^–N Removal Function of Initial NH_4_^+^–N Concentration

**Figure 5:**
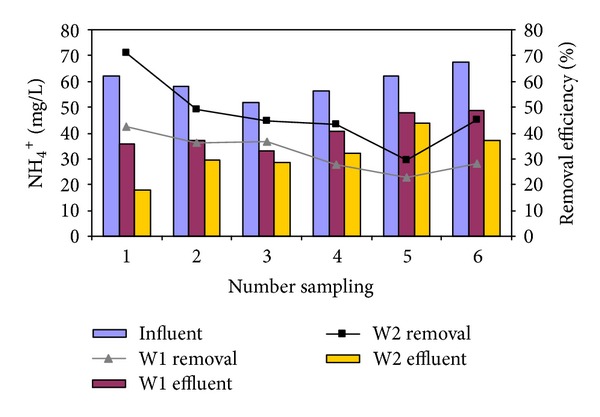
Influence of initial NH_4_^+^–N concentration on effluent and removal efficiency of NH_4_^+^–N in W1 and W2 (HRT: 42 d and 16.5°C).

The efficiency of *Cyperus alternifolius* for NH_4_^+^–N removal was assessed in NH_4_^+^–N concentration of 62.3, 58.3, 52, 56, 5, 62, and 67.5 mg/L corresponding to NH_4_^+^–N loading of 0.31, 0.29, 0.26, 0.28, 0.31, and 0.34 g/m^2^.d, respectively.  Figure 5 gives the NH_4_^+^–N removal percentage in both reactors function of initial NH_4_^+^–N concentration. The results are depicted in Figure 5, which shows a decline in the NH_4_^+^–N removal efficiency with rising initial NH_4_^+^–N concentration. It was found that the NH_4_^+^–N removal efficiency by W1 and W2 diminished from 43% and 71% to 28% and 45% when the NH_4_^+^–N concentration was amplified from 62 to 68 mg/L, respectively.

As seen in Table 4, in the initial NH_4_^+^–N concentration 62 to 68 mg/L, the W2 showed a low effluent NH_4_^+^–N concentration in comparison with the W1; however, the difference was statistically significant (*P* value > 0.05). Relatively high correlation was not observed between initial NH_4_^+^–N concentration and removal percentage of NH_4_^+^–N (*r*: −0.026, *P* value > 0.93).

## 4. Discussion

The study found that the highest efficiency of wetland in wastewater treatment was related to the elimination of COD, which increased at the presence of *Cyperus alternifolius*. The lowest efficiency was seen for Phosphorus elimination which did not show a significant change at the presence of *Cyperus alternifolius *(9.8%). The cause of little elimination of phosphorus in the control reactor is phosphorus absorption by the soil, biological elimination by existing bacteria, and complex formation and sediment at the presence of calcium, iron, and magnesium [10, 14]. The amount of phosphorus removal in similar systems that used *Cyperus alternifolius* was reported as 83.2% and in another study was less than 20% [10, 14, 15]. As shown in Figure 4, in some points, the amount of outlet phosphorus is more than the inlet phosphorus (negative elimination efficiency), which is caused by the release of phosphorus. In some other studies, the release of phosphorus was reported during the saturation of soil or during the reduction of incoming phosphorus. Another reason behind the increase in the outlet phosphorus in the main reactor in this study could be the death of *Cyperus alternifolius* and the release of the phosphorus from the pieces of the plant [14]. The amount of eliminated ammonium (NH_4_^+^–N) in the main reactor is 43%, which had 10% rise compared to that of the control reactor. An elimination amount of 75.3%, 70%, and less than 20% has been reported for ammonium (NH_4_^+^–N) [10, 15]. The processes helping the elimination of ammonium are the absorption by the plant and bacterial nitrification/de-nitrification [10]. In various studies, the efficiency of wetlands in the elimination of ammonium (NH_4_^+^–N) has been reported differently, even as little as 1% [14]. One mechanism for ammonium (NH_4_^+^–N) removal is volatilization, that happened in pH > 9; thus this mechanism effect is minimal in this study (pH in this study: 8.5) [10]. Cui et al. (2009) found that in China *Cyperus alternifolius* played a considerable role in eliminating TN (total nitrogen) from the domestic wastewater in artificial wetland with a vertical current [16]. Liao et al. studied the ability of *Cyperus alternifolius* and Vetiver in treating the pig farm wastewater. The efficiency of the BOD, COD, NH_4_^+^–N, and TP (total phosphorus) removal in this study was 68%, 64%, 20%, and 18%, respectively [9]. In another study, the ability of *Cyperus alternifolius *in elimination of nutrients including nitrogen, phosphorus, copper, and zinc from wastewater was reported to be from 4 to 7 times more than that of *Vetiveria zizanioides *plant [17]. The COD removal in the main reactor was very high. This high level was maintained during the study and only a small decrease was observed near the end of the study. The decrease may have been caused by the death of the plants. This was going to decrease with phosphorus rise in outlet samples, which supported the death of the plant at this stage [14].

## 5. Conclusion

As a matter of fact, using *Cyperus alternifolius* is capable of eliminating such parameters as COD properly. When primary treatment of wastewater is essential, application of this plant is very helpful and produce good results. However, the same system is not appropriate for advanced wastewater treatments; that is, this system is not helpful for tertiary purposes and nutrients removal such asphosphorus. This could be because of local conditions. Therefore, it is recommended to use *Cyperus alternifolius* with another plant that is well capable of phosphorus elimination. It is also suggested that outgoing wastewater be treated in a separate stage through chemical techniques for phosphorus eliminating. Overall, *Cyperus alternifolius* has been widely used in wetlands because of its cost-effectiveness; it can also be used as forage for livestock and aquaculture, and it is cost-effective due to fast growing.

## Figures and Tables

**Table 1:** Comparison of effluent concentration and removal efficiency of COD by each reactor.

Reactor	Effluent			Removal		
	Mean	SD	*t*-test	Mean	SD	*t*-test
W1	124.67	27.23	0.004	72.15	6.72	0.007
W2	75.5	18.33		83.15	4.45	

**Table 2:** Comparison of effluent concentration and removal efficiency of NO_3_-N by each reactor.

Reactor	Effluent			Removal		
	Mean	SD	*t*-test	Mean	SD	*t*-test
W1	2.15	0.58	0.52	86.22	5.68	0.75
W2	3.27	2.57		76.15	26.22	

**Table 3:** Comparison of effluent concentration and removal efficiency of PO_4_^−3^-P by each reactor.

Reactor	Effluent			Removal		
	Mean	SD	*t*-test	Mean	SD	*t*-test
W1	5.29	1.38	0.59	1.17	9.29	0.11
W2	4.81	1.55		11.41	10.91	

**Table 4:** Comparison of effluent concentration and removal efficiency of NH_4_^+^-N by each reactor.

Reactor	Effluent			Removal		
	Mean	SD	*t*-test	Mean	SD	*t*-test
W1	40.5	6.42	0.068	32.36	7.22	0.039
W2	31.5	8.65		47.19	13.53	
